# The Relationship Between Chinese English Major Students' Learning Anxiety and Enjoyment in an English Language Classroom: A Positive Psychology Perspective

**DOI:** 10.3389/fpsyg.2021.705244

**Published:** 2021-07-16

**Authors:** Fan Fang, Xiaofei Tang

**Affiliations:** Department of Foreign Languages and Literature, College of Liberal Arts, Shantou University, Shantou, China

**Keywords:** English language learning, foreign language enjoyment, foreign language classroom anxiety, higher education, second language acquisition

## Abstract

Many studies have explored learner psychology in relation to second language acquisition (SLA) in order to understand the effectiveness and difficulties of language learning. In the last two decades, emotional factors in students' language learning have garnered much attention in the field of SLA. However, more recently, studies have begun to focus on enjoyment and its relationship with anxiety. By collecting data at a provincial key university in southeast China, the study discussed in this paper investigated English major university students' emotions related to learning English. By collecting questionnaire responses from 140 English major undergraduates and conducting interviews with six students, the findings revealed that the participants' levels of foreign language enjoyment (FLE) were significantly higher than their levels of foreign language classroom anxiety (FLCA) and that they experienced FLE more frequently than FLCA. It was also found that the participants' FLE was more related to their teachers and peers and their FLCA was more related to their emotions, such as fear of a negative evaluation and speaking without sufficient preparation. In addition, this study also provides a few pedagogical implications for improving foreign language learning outcomes and teaching efficiency in English teaching and learning.

## Introduction

There have been a number of advancements in the field of second language acquisition (SLA) that have created an understanding of the manner in which individual differences and social environments enable certain learners to more easily acquire a foreign language as compared to others (Horwitz and Young, 1991; Dörnyei, 2009; MacIntyre and Gregersen, 2012; Dewaele and MacIntyre, 2014). For example, the concept of individual differences in language learning has expanded to the psychological aspects and social experiences of learners to “elaborate on the interplay among language, agent, and environment” (Dörnyei, 2009, p. 231). Among these individual differences, affective factors have become a key research focus in recent decades to understand learning anxiety, motivation, personality, and the various social aspects that affect language learning. The study discussed in this paper viewed learner anxiety and enjoyment as two closely linked psychological factors that are relevant to the external learning environment in order to unpack the classroom practice sessions and learning outcomes of second language (L2) learners.

While the psychological aspects of learner anxiety in SLA have been widely explored (Horwitz and Young, 1991; Horwitz, 2001; Dewaele and Al-Saraj, 2015; MacIntyre, 2017), the positive side of emotion—foreign language enjoyment (FLE)—has only recently drawn the attention of researchers (MacIntyre and Gregersen, 2012; Dewaele and MacIntyre, 2014, 2016; MacIntyre and Mercer, 2014; Dewaele et al., 2016; Elahi Shirvan and Taherian, 2020; Elahi Shirvan et al., 2021). Researchers have also begun to investigate the relationship between foreign language classroom anxiety (FLCA) and FLE (Dewaele and MacIntyre, 2014, 2016; Jiang and Dewaele, 2019). Although both FLE and FLCA affect the process of foreign language learning, learners might demonstrate different levels of FLE and FLCA. Therefore, a more holistic understanding of the relationship between FLE and FLCA needs to be further explored in various contexts.

In the Chinese context, for example, the emotions that foreign language learners experience in the classroom may be different from those of learners in other parts of the world (Jiang and Dewaele, 2019). The present study adopted both the Chinese Foreign Language Enjoyment Scale (CFLES) (Li et al., 2018) and the Foreign Language Classroom Anxiety Scale (FLCAS) (Horwitz et al., 1986) to explore English major students' emotions related to learning in English language classrooms. It is hoped that this study contributes to a further understanding of FLE and FLCA for English major students in the Chinese context, and offer some pedagogical implications for other English as a foreign language (EFL) contexts. In particular, this study aimed to provide language practitioners with suggestions to better understand the relationship between students' FLE and their FLCA. It is also hoped that this study contributes to deepening the understanding of the various needs and goals of students engaged in the L2 learning process. Further, it is also intended that some of the implications can help language practitioners modify their teaching strategies and class design to help students learn English more efficiently and sufficiently by reducing the students' FLCA and boosting their FLE.

## Literature Review

### Understanding Foreign Language Anxiety and Enjoyment

MacIntyre (1999) defined foreign language anxiety (FLA) as “the worry and negative emotional reaction aroused when learning or using a second language” (p. 27). Horwitz (2017) emphasized that the concept of anxiety is multi-faceted, as foreign language learners experiencing FLCA “have the trait of feeling state anxiety when participating in language learning and/or use” (p. 33). As a negative emotion, studies on SLA have primarily investigated anxiety in order to understand its characteristics and functions in the process of language learning (MacIntyre, 1999; Dewaele and Dewaele, 2017; Horwitz, 2017). FLA has been described as one of the strongest predictors of success or failure in foreign language learning (MacIntyre, 1999; Teimouri et al., 2019; Botes et al., 2020). Previous studies have indicated that FLA negatively affects the learners' language learning and communication skills (Dewaele, 2002; Gregersen and MacIntyre, 2014). It has also been demonstrated that anxiety can disrupt language acquisition in the stages of input, processing, and output (MacIntyre and Gardner, 1994).

Although FLCA has been well-researched from the 1980s onward, scholars have called for a more holistic understanding of foreign language classroom emotion for cognitive and motivational benefits as well as learning well-being (Dewaele and MacIntyre, 2014; MacIntyre and Vincze, 2017; Li et al., 2018). Recently, the research focus has shifted from investigating negative emotions to studying both negative and positive emotions (Dewaele and MacIntyre, 2014; Elahi Shirvan et al., 2020; Li, 2020; Elahi et al., 2021). The concept of “positive psychology” was first introduced to the field of SLA by MacIntyre and Gregersen (2012). Based on Fredrickson's (2003) broaden-and-build theory, MacIntyre and Gregersen (2012) argued that positive emotions can “broaden people's momentary thought-action repertoires and build their enduring personal resources, ranging from physical and intellectual resources to social and psychological resources” (p. 5). In the context of language learning, positive emotions are much more than pleasant feelings—enjoyment can be summarized as the good feelings that arise from breaking through homeostatic limits and stretching beyond one's limitations to accomplish something new or even unexpected, particularly when facing difficult tasks (Csikszentmihalyi, 2014). Learners in the grip of positive emotions are better able to notice things in their classroom environment and become more aware of language input. Thus, they can better attend to, process, and acquire a target language (Li et al., 2018; Saito et al., 2018). Moreover, positive emotions have long-term effects beyond the classroom, as they make students more resilient during difficult times (Dewaele and MacIntyre, 2019; Li, 2020).

### Studies on the Relationships Between FLE and FLCA

Dewaele and MacIntyre (2014) were the first to explore the relationship between FLE and FLCA. In their study, which adopted a mix-methods approach, 1,746 participants reported significantly higher levels of FLE than FLCA, showing “consistently high levels of enjoyment with less variation around the mean than for FLCA” (p. 261). A significant negative correlation was found between the FLE and FLCA of foreign language learners of all ages worldwide. More advanced learners who felt that they did better than their peers in foreign language classes reported significantly higher levels of FLE and significantly lower levels of FLCA. In addition, classroom activities—such as role-playing, games, debates, group presentations, a good classroom environment, and positive teachers with a good sense of humor—all played important roles in boosting the learners' levels of FLE.

Dewaele and MacIntyre (2016) conducted a follow-up study in which they applied principal component analysis to the same data set. While the participants were found to have significantly higher levels of FLE than FLCA, the narrative analysis revealed that female students reported having more fun in their foreign language classroom than male students. The study revealed specific causes of FLE, such as good marks to increase the students' pride, determination to excel in the foreign language class, collaborative learning to strengthen their social bonds, and the use of fun and interesting games in the classroom. They identified two sub-dimensions of FLE: FLE-Social and FLE-Private. A follow-up study by Jiang and Dewaele (2019) defined FLE-Social as “reflected in shared legends, classroom laughter, and friendly relationships with teachers and peers” (p. 15), while FLE-Private is defined as “reflected in internal feelings, such as pride, having fun, and a sense of achievement” (p. 15).

Dewaele et al. (2018) explored the effect of learner-internal and learner-external variables on the FLE and FLCA levels of secondary school pupils in London. They found significantly higher levels of FLE than FLCA, with a weak negative correlation between both. Further, higher levels of FLE were additionally related to more positive attitudes toward the foreign language, the foreign language teacher, frequent use of the language by the teacher in class, and the amount of time the pupils practiced speaking the L2. FLE was also found to have a greater positive impact on teachers and teaching practices than FLCA. Another study by Dewaele and Dewaele (2017) with the same database of secondary school pupils utilized a pseudo-longitudinal design to investigate how FLE and FLCA evolved among pupils in different age groups. The results revealed a slight increase in FLE among three age groups and little variation in FLCA; however, the weak negative correlation between FLE and FLCA remained constant over time. Another study by Dewaele and Dewaele (2020) that investigated two teachers based in London found a significantly higher FLE for the main teacher than the second teacher, while no difference was found in the FLCA for these two teachers. Elahi et al. (2021) investigated the increase in and changing trends of FLE and FLCA in a general English course. They found that while the participants' FLE increased significantly during the semester, their levels of FLCA decreased. Moreover, the initial levels of both FLE and FLCA were not indicative of whether these would increase during the semester. Further, Jin and Zhang (2018) investigated 320 EFL learners' foreign language classroom enjoyment and revealed a three-factor solution for an FLE—*Enjoyment of Teacher Support, Enjoyment of Student Support*, and *Enjoyment of Foreign Language Learning*. A key finding from the study is that *Enjoyment of Foreign Language Learning* had a direct effect on students' mid-term scores. Li (2020) explored the complex relationships between 1,307 Chinese high school students and found a low to moderate level of FLE among the students.

### Researching FLE and FLCA in the Chinese Context

In China, there remains a scarcity of research on positive emotions in relation to foreign language learning (Liu and Jackson, 2008; Jin et al., 2015; Li et al., 2020). More recently, scholars have begun to explore positive emotions in foreign language learning. Li et al. (2018) conducted the first systematic investigation of FLE in a cohort of Chinese high school students. The CFLES, the Chinese version of the FLES, was developed with explanations of its psychometric properties. Three dimensions of the CFLES were identified: FLE-Private (private pleasure related to personal progress, excellent performance, or interesting experiences in EFL learning), FLE-Teacher (enjoyable experiences related to the teachers' supportive and encouraging attitude toward students and their pedagogical practices), and FLE-Atmosphere (classroom group activities that increase the students' levels of FLE). The results revealed that the students' FLE-Teacher level was the highest, followed by FLE-Private and FLE-Atmosphere. Their findings also revealed that an individual student's experience of FLE originated from a wide range of learner-internal and learner-external variables, such as the learner's self-realization, the teacher, the peer group, and the classroom environment.

Li et al.'s (2020) study adopted a complex dynamic systems theory approach, which focuses on the link among FLE, FLCA, and EFL achievement in Chinese EFL students. FLE and FLCA were found to be unique predictors of self-perceived EFL proficiency at various achievement levels. The relationship was stronger in the group with a higher level of achievement and weaker in the group with a lower level of achievement. An analysis of the qualitative data revealed that low scores in English tests and fear of teacher criticism were major sources of FLCA; on the other hand, good test results, teacher praise, teacher support and increased social standing in the group were the main sources of FLE (De Ruiter et al., 2019).

Jiang and Dewaele (2019) investigated the uniqueness of the emotions that Chinese foreign language learners experience in the classroom and how cultural factors, such as the role of the teacher in the Chinese educational context, could shape the manner in which interactions between the learner-internal and learner-external variables impacted FLE and FLCA. They identified several dimensions in relation to FLE and FLCA, including FLE/FLCA-Self, FLE/FLCA-Teacher, and FLE/FLCA-Peer. Among these, the participants' FLE was primarily related to the teacher and teaching practices. The participants' FLCA was mostly related to their personal level of anxiety, followed by FLCA-Teacher and FLCA-Peer. In that study, the participants reported similar levels of FLE but higher levels of FLCA in comparison to the international sample in Dewaele and MacIntyre's (2014) study. Further, multiple regression analyses revealed that FLE was predicted more strongly by teacher-related variables, while FLCA was primarily predicted by learner-internal variables. With the call for a more holistic understanding of foreign language emotion for students with specific subject domains, the present study aimed to investigate the relationship between English major students' FLE and FLCA from the perspective of positive psychology.

## Methodology

### Research Questions

The present study aimed to explore the relationship between FLE and FLCA among English major students and the levels of both aspects in this group of students (cf. Dewaele et al., 2019). It was hypothesized that the results will likely reflect the current situation and status of English major students' anxiety and enjoyment while learning the English language and offer a few suggestions for strengthening English language teaching and learning. The study aimed to address the following research questions:

1. What are the levels of FLE and FLCA perceived by English major students?
2. Is there a significant correlation between FLE and FLCA among English major students from four instructional levels?
3. What do English major students perceive as being the possible reasons, if any, for their FLE and FLCA?

### Setting and Participants

This research was conducted at a provincial key university located in southeast China. The university has over 10,000 students and approximately 300 students major in English in the College of Liberal Arts. This group of students specialize in Chinese tertiary education because they are learning EFL as well as researching language and culture, translation, literature, linguistics, and applied linguistics for their major and subject studies. Thus, their learning experiences deserve additional attention. Second, at the meso- and micro-levels, the first author currently works at the university where English proficiency is emphasized. As a teacher in the foreign language department, the first author conveyed a few challenges shared by the students regarding their learning struggles and (de)motivation during their learning journeys as well as a few joyful moments of success and achievement. Therefore, we believe that it was necessary to investigate English major students' FLE and FLCA in order to supplement the existing knowledge of SLA and foreign language learning because of the specific learning needs and objectives of this group of students. This study echoes some previous studies that investigate university students in general (Li et al., 2018, 2020; Jiang and Dewaele, 2019) to explore English major students' FLE and FLCA in Chinese tertiary education.

In the first phase of this study, 140 English major undergraduates completed the questionnaire. The participants were aged from 18 to 23 years (M = 20 years, SD = 1.5) and included 47 first-year students, 32 second-year students, 30 third-year students, and 31 fourth-year students. Due to the nature of English major students in China with more female students enrolled, the questionnaire respondents included 24 male students (17.1%) and 116 female students (82.9%). Six volunteers left their contact information and were selected to participate in follow-up interviews (for more detailed information, see Table 1). They were selected from among the students who had completed the questionnaire and the second author contacted them via WeChat, a popular social media application among Chinese people. After being provided with information regarding the content and purpose of the interview, they all agreed to attend the interview session.

**Table 1:** Information about the Interviewees and the Length of the Interviews.

**Interviewee**	**Gender**	**Grade level**	**Age**	**Length of the interview**
S1	Female	First Year	19	25:22
S2	Female	Second Year	20	26:06
S3	Female	Second Year	20	25:31
S4	Male	Third Year	20	28:26
S5	Female	Third Year	21	26:15
S6	Female	Fourth Year	22	27:06

### Instrumentation

The study utilized a questionnaire and a series of semi-structured interviews to collect data. The questionnaire consisted of the CFLES (Li et al., 2018) and the FLCAS (Horwitz et al., 1986), with a total of 42 items (see Appendix). In each of the items, responses were given based on the standard five-point Likert scale (ranging from strongly disagree = 1 to strongly agree = 5). This version of CFLES contains 11 items that indicate 3 dimensions: FLE-Private, FLE-Teacher, and FLE-Atmosphere. As the CFLES has been validated among a large-scale sample (Li et al., 2018), we included all the items in this study. With regard to items related to FLCAS (Horwitz et al., 1986), the term *foreign language* was changed to *English language* in order to study the classroom anxiety of English major students in major-related courses that the students took during the school year. We selected 31 items of the FLCAS because the students did not take classes that were lectured by native speakers during the year in which data was collected^1^. Therefore, the FLCAS in this study contained 31 items that indicate four dimensions: communication apprehension, test anxiety, fear of negative evaluation, and other anxieties. The Cronbach's Alpha was 0.828, thereby indicating good internal consistency reliability for the adapted scale.

As previously noted, six students were recruited after they expressed an interest in participating in follow-up interviews when completing the questionnaire. The semi-structured interviews served as a tool to provide possible explanations for the results of the questionnaire (Kvale, 2008) and to better understand the reasons for the students' FLE and FLCA and the correlation between them. Echoing Dewaele and MacIntyre (2014) and Li et al. (2018), we used two open-ended questions and asked the students to describe a specific event in their major courses in which they experienced FLE and FLCA: They were asked to describe one of the most enjoyable and one of the most anxious learning experiences they had in their English classes in that current year and to share their feelings about those experiences. They were also asked to provide a few suggestions for improving their learning experiences in future English language teaching contexts in order to enhance their positive feelings related to learning the language. This method aimed to acknowledge the sources of the students' FLE and FLCA and to offer practical advice to improve their learning in an English language classroom.

### Data Collection and Analysis

Data collection for this study was divided into two phases. In the first phase, the second author administered the questionnaire online in December 2019 to English major students from four instructional levels (first-year students through fourth-year students). The questionnaire was distributed through WeChat groups and was bilingual (in Chinese and English) in order to facilitate the participants' ability to better understand the items. While Li et al.'s (2018) questionnaire on FLE was already bilingual and utilized in this study, the original English version of the FLCA questionnaires was first translated by the researchers and then the translated items were checked by a professor specializing in translation studies in order to maintain the accuracy of translation. Further, the participants were told that all the information and answers would remain anonymous and confidential.

In the second phase, the interviews with the six students were also conducted online. The students were told that the interview questions were based on the questionnaire they had previously completed; moreover, they were informed in advance that the interview would be audio-recorded and saved only for research purposes. The interviews were conducted in Mandarin in order to enable the participants to respond to the questions in greater depth (Mann, 2011). All the recordings were transcribed verbatim by the authors, and the transcripts were returned to the interviewees for peer review.

**Figure 1:**
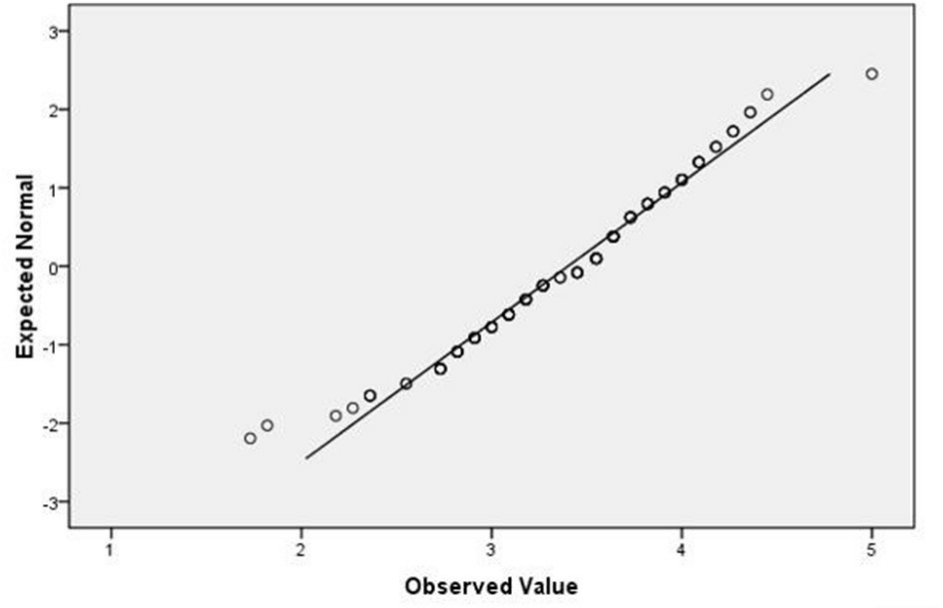
Normal Q-Q Plot of FLE.

**Figure 2:**
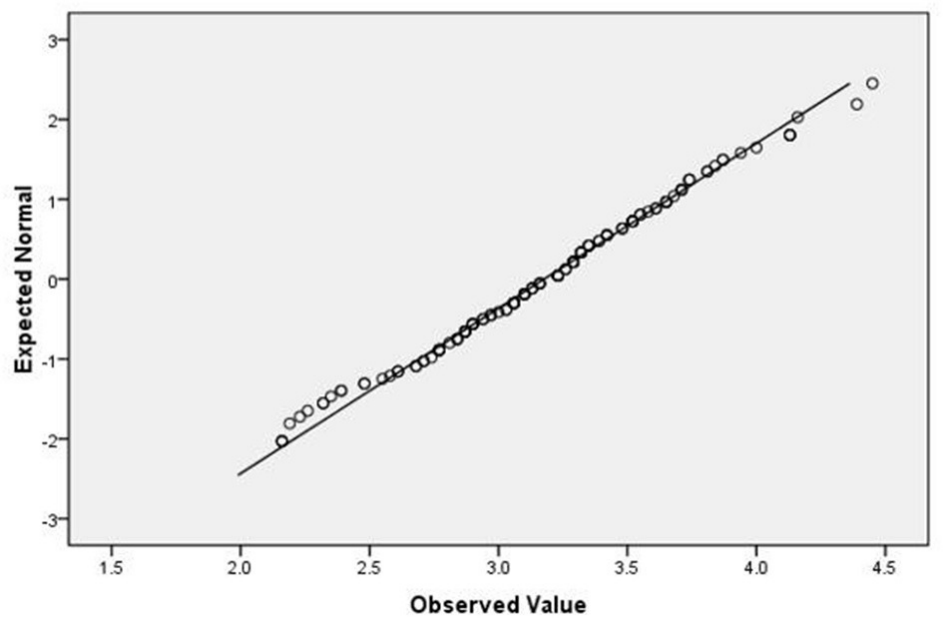
Normal Q-Q Plot of FLCA.

The questionnaire data were processed and analyzed using Statistical Package for the Social Sciences (SPSS) version 16.0. First, the mean scores of FLE and FLCA and their sub-scales were calculated. Then, a paired *t*-test of FLE and FLCA was conducted to see which scored higher among the participants. A scatterplot graph and Pearson's correlation were used to explore the correlation between FLE and FLCA. A close examination of the distribution of FLE and FLCA and the calculation of Q-Q plots (see Figures 1, 2) suggests that they reasonably follow a normal distribution. This has been confirmed by the skewness and kurtosis statistic values (FLE: skewness = −0.578, kurtosis = 0.358, *SE* = 0.205; FLCA: skewness = −0.077, kurtosis = −0.603, *SE* = 0.407). Then, a one-way analysis of variance (ANOVA) test was conducted to identify the differences between the means of the FLE and FLCA for students from the four instructional levels who had answered the second research question.

Further, qualitative content analysis (Schreier, 2012) was adopted to analyze the information obtained from the interviews “to explore the deeper meanings so as to add interpretive depth and breadth to the analysis” (Jenkins, 2014, p. 128) and to systematically organize the data. The data were analyzed by NVivo 11 to identify the key themes for the different dimensions of FLE and FLCA (cf. Li et al., 2018). The authors conducted the initial coding based on the research questions. Then, the second author conducted a second coding to group the interview data based on the data-driven themes. The first author conducted a final interpretative level of coding, drawing together the previous themes and research questions. Finally, three FLE categories were adopted: FLE-Teacher, FLE-Peer, and FLE-Self (cf. Jiang and Dewaele, 2019). These categories reflected the three dimensions that impact FLE and were consistent with the data that were collected. Further, the participants' interview data regarding FLCA were divided into three dimensions: fear of negative evaluation, test anxiety, and communication apprehension (cf. Horwitz et al., 1986).

## Findings

### Questionnaire Results

The questionnaire data aimed to answer the first and second research questions: “What are the levels of FLE and FLCA perceived by English major students?” and “Is there a significant correlation between FLE and FLCA among English major students from four instructional levels?”

The average scores (on the five-point scale) were calculated for FLE (*N* = 140, *M* = 3.4, *SD* = 0.31) and FLCA (*N* = 140, *M* = 3.2, *SD* = 0.23). The results revealed that English major students had a moderate level of FLE and FLCA. The average scores for the three FLE dimensions were also calculated: FLE-Teacher (*M* = 3.95, *SD* = 0.37), FLE-Atmosphere (*M* = 3.29, *SD* = 0.51), and FLE-Private (*M* = 3.01, *SD* = 0.47). The paired *t*-test revealed that the participants' reported significantly more enjoyment than anxiety when learning English in their current year's English classes (*df* = 139, *t* = 3.36, *p* < 0.001, *Cohen's d* = 0.73).

Further, the average scores for the four FLCA dimensions were also calculated: fear of negative evaluation (*M* = 3.21, *SD* = 0.26), test anxiety (*M* = 3.18, *SD* = 0.21), communication apprehension (*M* = 3.08, *SD* = 0.29), and other anxieties (*M* = 3.03, *SD* = 0.27). The results indicated that English major students had the highest level of FLCA for fear of negative evaluation, followed by test anxiety, communication apprehension, and other anxieties.

The scatterplot graph reveals that there was no significant correlation between the students' FLE and FLCA. The result of Pearson's correlation further confirmed that no correlation existed between the English major students' FLE and FLCA (*r* (140) = −0.135, *p* > 0.05).

The one-way ANOVA test revealed a significant difference between the means of the FLE scores (*F* = 5.11, *p* < 0.05, *eta*^2^ = 0.081, *Cohen's d* = 0.76) for the four instructional levels; however, there was no significant difference between the means of the FLCA scores (*F* = 1.16, *p* > 0.05, *eta*^2^ = 0.058, *Cohen's d* = 0.43). The FLE scores (*M* = 3.07) were lower for the fourth-year students than the students in the other instructional levels; the scores for each of the three dimensions were FLE-Teacher (*M* = 3.58), FLE-Atmosphere (*M* = 3.02), and FLE-Private (*M* = 2.82). The FLE scores (*M* = 3.55) were higher for the third-year students than the students in the other instructional levels; the scores for each of the FLE dimensions were: FLE-Teacher (*M* = 4.06), FLE-Atmosphere (*M* = 3.42), and FLE-Private (*M* = 3.27). Thus, the results indicated that students from different instructional levels experienced different levels of FLE and FLCA. *Post-hoc* Tukey tests revealed that fourth-year students scored significantly lower in FLE than other grades (all *p* < 0.05) and no significant difference for FLCA scores was found among all four grades (all *p* > 0.05).

### Interview Findings

The interviews were conducted to answer the third research question: “What do English major students perceive as being the possible reasons, if any, for their FLE and FLCA?” The findings from the interviews are presented in relation to the three main FLE themes and the three FLCA dimensions.

#### Sources of FLE

As previously noted, the sources of FLE can be classified into three dimensions: FLE-Teacher, FLE-Peer, and FLE-Self (Jiang and Dewaele, 2019). For FLE-Teacher, the teacher was mentioned as the student's direct source of enjoyment. For FLE-Peer, the behavior of a student's peers or the student's interaction with peers were the direct source of enjoyment. For FLE-Self, the participants themselves were the primary source of enjoyment.

##### FLE–Teacher

For the FLE-Teacher dimension, specific classroom activities were the source of enjoyment that was most often mentioned by the participants; this was followed by teacher's friendliness and using humor to teach language skills and knowledge. The participants mentioned that novel, interesting, and meaningful classroom activities—such as role playing, debates, and watching class-related videos—led to effective learning. For example, S3 mentioned an enjoyable experience in her class in relation to learning about Chinese culture:

**Extract 1**

Once, our teacher invited her friend who is an expert on pottery. He not only first introduced us to basic knowledge about pottery but also taught us how to make pottery with real materials and tools. I felt so happy because I gained this knowledge and I also put it into practice, which brought me a sense of involvement and achievement.

This extract reveals that students found a sense of enjoyment if learning was not restricted to content in a textbook. Certain activities organized by teachers also aroused students' interests, which could be a source of FLE. Moreover, four of the students mentioned that their teachers' friendliness improved their enjoyment of learning English, which indicated that the teachers' personality played a key role as a source of the students' FLE. For example, S5 described how the teacher encouraged her and helped her learn English in the class:

**Extract 2**

During our group discussion, the teacher would walk around, listen to, and participate in our discussion. He encouraged us to share our ideas with our peers and with him. He would give us feedback instantly, in a friendly way, just like talking to friends.

Further, four students mentioned that both the teachers' personal charisma and the teaching materials were vital for making them feel involved in class and enjoy the process of learning English. This is evident from the following extract from S4:

**Extract 3**

The teacher is very interesting and knowledgeable. He knows what attracts his students and how to teach us theoretical knowledge in an understandable and interesting way. He was always absorbed and passionate when reading English poems, which impressed me a lot.

The above extracts indicate the importance of the teacher's role in facilitating the students' FLE. Therefore, it is essential for teachers to understand their role in order to ensure that their teaching methods have a positive influence on their students.

##### FLE–Peer

For the FLE-Peer dimension, interactions with peers were most often mentioned as the source of enjoyment, along with the friendliness and positive attitudes of peers. This is evident from the following statement made by S2:

**Extract 4**

I was really happy when our group worked together to prepare for some class activities and homework. In this process, we exchanged our ideas and helped each other.

Further, two participants mentioned delightful interactions with their peers. For example, S1 mentioned that peer interaction brought her happiness and stimulated her passion for learning English in class:

**Extract 5**

In my group, most of the members were very outgoing and humorous. They were brave enough to express their ideas in English. Their interesting and novel thoughts were so affective that inspired me a lot. They were good at livening the atmosphere and making all the group members stay focused and excited.

These extracts indicate that peers can also enhance a student's FLE. Unlike teachers, students spend more time with their peers on campus. Such positive effects within and out of the classroom also created a long-term FLE for the students.

##### FLE–Self

For the FLE-Self dimension, the participants often enjoyed themselves when they performed well in class. S6 described how excited and happy she was when she answered the teacher's question well:

**Extract 6**

When the teacher asked the whole class a question related to the last lesson, no one except me uttered the right answer. The teacher felt happy and praised me, saying, “Very good”. I also felt a sense of happiness in that moment. Even after a long time, when I thought of that scene, I felt quite happy.

In addition, positive experiences and a sense of enjoyment were mentioned by five of the participants. One key reason was that these participants were all English majors who were highly motivated to learn. It is understandable that they felt a sense of enjoyment when they performed well in class and were praised by the teacher.

#### Sources of FLCA

In terms of the sources of FLCA, the interview data showed that the English major students primarily experienced three types of anxiety: fear of negative evaluation, test anxiety, and communication apprehension. This is consistent with the dimensions of the FLCAS (Horwitz et al., 1986). While three types of FLCA were analyzed in our study, these types (or “factor models”) are not independent; rather, they are interconnected, because language learning is complicated and multifaceted and it transcends the various types of FLCA (Horwitz, 2017). However, it is necessary to identify the types of FLCA before language teachers can view language learning as a whole process and, thus, develop and implement “more targeted anxiety-reduction procedures” (Horwitz, 2017, p. 38).

Among the three types of anxiety, FLCA was most related to fear of negative evaluation and test anxiety than communication apprehension. Being called upon to answer questions and being afraid of making mistakes in class were the sources of FLCA that were most frequently reported, followed by taking a test in class, lacking preparation before class, and difficult learning materials.

##### FLCA: Fear of Negative Evaluation

In the dimension of the fear of negative evaluation dimension, most of the students mentioned that they were afraid of making mistakes in front of the teacher and their classmates. As shown in the extracts below, both S1 and S3 described their most anxious experience:

**Extract 7**

I was not prepared for that class, and I was ordered to answer a question. My brain went blank, and I felt so embarrassed. I didn't speak a word until the teacher helped me say the former part and I answered the latter part in a soft voice.

**Extract 8**

I am very anxious every time when I have to do a presentation in front of my classmates and the teacher. Once, I mispronounced a word, and I became more nervous that I could not even speak fluently later.

It was found that this type of anxiety is related to the Chinese cultural aspect of “saving face” during the learning process. These cases revealed that the students were afraid of losing face in front of their teacher and peers in case they provided wrong answers, thereby generating anxiety. Thus, the relationship among saving face, identity negotiation, and the level of anxiety must be recognized when students are learning a foreign language (Jin and Cortazzi, 2011; Li and Ma, 2018).

##### FLCA: Test Anxiety

The participants also mentioned test anxiety in relation to the need to take frequent tests in class. Frequent or unexpected tests in class were found to greatly aggravate the students' anxious feelings, which is evident from the following statement by S6:

**Extract 9**

In our class of prose and fiction, I used to feel anxious before class because we would have a quiz about new vocabularies, sentences, and some phrases that we learned before.

##### FLCA: Communication Apprehension

In terms of communication apprehension, communicating with teachers in front of classmates was the most frequently mentioned source by students. The students felt anxious when they conversed unsuccessfully with their teachers in the classrooms. This is evident from the following statements made by S2:

**Extract 10**

When conversing with the teacher in class, I answered the teacher's question, but the teacher could not understand what I said. I explained again, yet she still did not understand. Then, I got very embarrassed in that moment.

It must be noted that, similar to the fear of negative evaluation, the position of the teacher as the authority in traditional Chinese education culture also likely makes students feel anxious about communicating with their instructors, particularly when experiencing a communication breakdown (Jin and Cortazzi, 2011; Deng, 2014).

The analyses of the possible sources of FLE and FLCA reveal that the triggers for FLE were most often linked to the teacher and the students' peers, while the triggers for FLCA most often originated within the students themselves and were related to the teacher as well, although other factors such as family backgrounds and interpersonal relationships might also contribute to students' FLCA. Moreover, among the three FLE dimensions, FLE-Peer was mentioned most frequently, followed by FLE-Teacher and FLE-Self, which concurs with the findings reported in previous studies (Jiang and Dewaele, 2019; Li et al., 2020). Among the three dimensions of FLCA, fear of negative evaluation was the most frequently mentioned cause, followed by test anxiety and communication apprehension, which is consistent with the analysis of the questionnaire data.

## Discussion and Implications

### Summary of the Findings of the Research Questions

The findings of this study provide a more in-depth understanding of Chinese English major students' levels of FLE and FLCA and of the reasons these emotions occur. The first research question examined the English major students' levels of FLE and FLCA. The participants reported relatively high levels of FLE and low levels of FLCA. Feelings of enjoyment were more prevalent than anxiety, which echoes the findings reported in previous studies (Dewaele and MacIntyre, 2014; Dewaele et al., 2018; Jiang and Dewaele, 2019). It should be noted that the factors contributing to FLE are complex and dynamic, but the students mainly enjoy English learning when they obtain teachers' encouragement, success in group discussion, and their own performance. It should be understood that both internal and external factors contributing to FLE play a role in students' English learning process. In the present study, the English major students reported higher levels of FLE (*M* = 3.4) than the Chinese high school students (*M* = 3.12) in Li et al.'s (2018) study. This indicates that the Chinese English major students in the focal university felt more FLE than the Chinese high school students.

Further, the participants reported a higher level of FLE for the FLE-Teacher dimension than the FLE-Private and FLE-Atmosphere dimensions. They also reported higher FLCA for fear of negative evaluation than for test anxiety, communication apprehension, and other anxieties. The results concur with the findings reported in Jiang and Dewaele (2019), thereby revealing that a teacher continues to play a prominent role in influencing students' emotions and in their language learning experience in the Chinese context. The collectivist culture of China also has a significant effect on changes in the students' emotions in a classroom. In addition, apart from teachers, peer-related experience can also contribute to FLE and FLCA. Therefore, the role played by both teachers and peers is important in creating a conducive learning environment for a positive learning outcome for students.

Unlike the results reported in previous studies (Dewaele and MacIntyre, 2014; Jiang and Dewaele, 2019), the present study found that there was no significant correlation between the English major students' FLE and FLCA. This finding might be due to the timeframe (1 year) employed as an instruction in the current study or the variations in the variables. For example, the participants in the present study were from different instructional levels and took a different number and type of courses and, thus, they might have encountered different teachers, classmates, and course requirements. Most of these variables have been proven to be closely related to learners' levels of FLE and FLCA (Dewaele and MacIntyre, 2014; Jiang and Dewaele, 2019); consequently, these variables might influence the final result.

The second research question dealt with differences in FLE and FLCA among English major students from four instructional levels. A significant instructional-level difference was found for FLE, but not for FLCA. The findings reveal that the students in the four instructional levels experienced different levels of FLE; the FLE score was lowest for the fourth-year students and highest for the third-year students. Further analysis revealed that the fourth-year students reported significantly lower levels of FLE-Private and FLE-Atmosphere than the students in the other instructional levels; moreover, the third-year students reported significantly higher levels of FLE-Atmosphere. This result might be related to how the courses were arranged and the learning experiences that the participants were processing. For example, the fourth-year students may have fewer courses and may pay more attention to finding a job or preparing for certification exams. Thus, they may feel less FLE in class. It is also possible that third-year students begin focusing on their future to determine whether to pursue postgraduate study or look for a job. The third year is regarded as the transition year in which students encounter various levels of hardship and dilemmas as well as opportunities and challenges. Thus, in order to enhance their learning enjoyment, students' emotional needs must be better understood to allay their learning anxiety and celebrate their success. Further, it is also important to investigate the design of courses and assessment measures throughout the four-year university studies in order to further communicate with students' to understand their enjoyment and struggle with course design and difficulty level of courses.

The third research question explored the sources of FLE and FLCA in the participants' experiences in their English classes. Peer-related experiences were the most frequently mentioned source of FLE and fear of negative evaluation was the most frequently mentioned source of FLCA. These findings confirmed the view that FLCA originates more from the learner, while FLE is more context-dependent and is linked to the behavior of the teacher and the students' peers and the interactions between them (Dewaele et al., 2018; Jiang and Dewaele, 2019). In addition, it must be noted that the sources of FLE and FLCA are multifaceted and are impacted by ideology and socioeconomic and sociocultural aspects (Fang, 2018). It is important for language teachers to realize that language learning is stressful, while “a search for facilitative anxiety [is] a *step backwards* and even a dangerous trend” (Horwitz, 2017, p. 40).

### Unpacking FLCA and FLE: Implications

The study's findings have specific pedagogical implications that will likely enable teachers and students to gain a better understanding of the relationship between multifaceted emotions and English language teaching and learning in the classroom. First, teachers are encouraged to be friendly, supportive, and humorous, as well as to implement novel and interesting classroom activities (Dewaele et al., 2018; Jiang and Dewaele, 2019; Li et al., 2020). They must also employ more interactive teaching activities to present the course contents in suitable ways based on their students' English proficiency and their needs and goals. The teacher's use of humor can stimulate the students' interests and increase positive learning emotions in an English language learning classroom (Fredrickson, 2003). Moreover, teachers are expected to be professional and present the content in a manner that is easy to understand. For example, foreign language teachers must be equipped with basic teaching skills, be familiar with the course contents, and be well-prepared. Since English major students demand more subject-specific knowledge of English, teachers with professional competence and a student-friendly approach are more likely to improve the level of the students' FLE. In addition, teachers can also provide adequate interactive and collaborative classroom activities, thereby creating a relaxing and positive environment. For example, in linguistics lessons, teachers can prepare certain topics for class discussion, divide students into groups, and then share or even “perform” their ideas through “drama-based learning techniques.” For example, when conducting pop-up quizzes, it might be helpful for language teachers to manage their difficulty level and determine how often they need to be administered. Lastly, although the English major students in this study reported having a relatively low FLCA, teachers must encourage students to answer questions voluntarily rather than directly calling on them.

On the other hand, students are encouraged to be well-prepared in order to reduce the anxiety associated with “being afraid of answering questions,” “making mistakes,” and “losing track of what is being taught.” Adequate preparations will boost the students' confidence and, to a certain extent, alleviate their FLCA (MacIntyre, 1999). Since FLCA has been found to have negative effects on language performance, students must actively participate in their learning in order to overcome their anxiety. In addition to preparing to learn a language, actively attending the class and maintaining a positive attitude toward learning English could also improve learning enjoyment. Further, students must be aware of the course requirements, teaching materials, and performance assessment. It is important that students develop a positive attitude toward language learning because they are attending universities to acquire knowledge and improve themselves. The more students' focus on knowledge acquisition, the less attention they pay to others' evaluations of their performance. This will enable them to feel happiness and a sense of fulfillment.

In addition, policymakers can adjust the course requirements and course design to reduce the pressure students feel and grant teachers more freedom to design their courses. For example, the most basic and compulsory courses must begin at the lower-intermediate instructional level. Students must be given the right to complete all the courses before advancing to the advanced level. The institution must also provide teachers more freedom to decide their own pedagogical practice and learning assessments and avoid requiring the same assessment for all classes, such as a fixed standard for attendance or tests and homework requirements for different courses. Such measures must also consider the gender issue, given the nature that female students outnumber male students in English major classes in the Chinese context. According to Jiang and Dewaele (2019), “To create a positive and relaxing language learning environment, the institution should reduce the frequency of exams and give the teacher more freedom in language assessment as well” (p. 22).

## Limitations and Conclusion

Before concluding, it must be noted that this study has a few limitations. First, it only examined the overt levels of students' FLE and FLCA within the specific year when they were completing the questionnaire. This time limit might have restrained the students from accurately reflecting on their FLE and FLCA, as the participants met different teachers and classmates when taking classes during the course of their university studies. Second, this study only included English major students from one university in China. However, while the findings of this study are not generalizable, it is possible that these findings may have some transferability to other settings (Lincoln and Guba, 1985). In the future, research may expand the sample when measuring the participants' FLE and FLCA to further reveal the correlation between FLE and language performance in different contexts.

Despite its limitations, this study is one of the first to investigate English major students' levels of FLE and FLCA in the Chinese context. This study also examined the differences between FLE and FLCA based on the students' instructional levels and the possible sources of their FLE and FLCA. The findings indicate that enjoyment was more prevalent than anxiety among the participants. It is hoped that the findings of this study can facilitate curriculum innovation and help improve students' English language learning performance. The findings are also expected to inspire the pedagogical practices of English language teachers and enrich the empirical research on foreign language education for English major students. Finally, further studies are needed to more fully focus on exploring the emotions of English major students in different contexts.

## Data Availability Statement

The datasets generated for this article are not readily available because we want to assure anonymity and confidentiality particularly for the interview data. Requests to access the datasets should be directed to Fan Fang, ffang@stu.edu.cn.

## Ethics Statement

The studies involving human participants were reviewed and approved by Shantou University Research Division. The patients/participants provided their written informed consent to participate in this study.

## Author Contributions

FF: supervision, funding, data analysis, and writing and revising of manuscript. XT: data collection, data analysis, and writing of manuscript. All authors contributed to the article and approved the submitted version.

## Conflict of Interest

The authors declare that the research was conducted in the absence of any commercial or financial relationships that could be construed as a potential conflict of interest.

## Footnotes

^1^These two items not included are “I would *not* be nervous speaking the foreign language with native speakers”; “I would probably feel comfortable around native speakers of the foreign language” (Horwitz et al., 1986, italics in original).

## Appendix Questionnaire

### A Survey of English Major Students' Learning Situation in English Classrooms

**Table:**

	**1**	**2**	**3**	**4**	**5**
	**Strongly Disagree**	**Disagree**	**Neutral**	**Agree**	**Strongly Agree**
1. I don't get bored in this year's English classes.	1	2	3	4	5
2. I enjoy this year's English classes.	1	2	3	4	5
3. I've learnt interesting things in this year's English classes.	1	2	3	4	5
4. In class, I feel proud of my accomplishments.	1	2	3	4	5
5. This year's English classes have a positive environment.	1	2	3	4	5
6. This year's English classes are fun.	1	2	3	4	5
7. The teachers are encouraging.	1	2	3	4	5
8. The teachers are friendly.	1	2	3	4	5
9. The teachers are supportive.	1	2	3	4	5
10. There is a good atmosphere.	1	2	3	4	5
11. We form a discussion group.	1	2	3	4	5
12. I never feel quite sure of myself when I am speaking in my English class.	1	2	3	4	5
13. I don't worry about making mistakes in English class.	1	2	3	4	5
14. I tremble when I know that I'm going to be called on in English class.	1	2	3	4	5
15. It frightens me when I don't understand what the teacher is saying in the English language.	1	2	3	4	5
16. It wouldn't bother me at all to take more English classes.	1	2	3	4	5
17. During English class, I find myself thinking about things that have nothing to do with the course.	1	2	3	4	5
18. I keep thinking that the other students are better at English than I am.	1	2	3	4	5
19. I am usually at ease when taking tests in my language class.	1	2	3	4	5
20. I start to panic when I have to speak in English class without being prepared.	1	2	3	4	5
21. I worry about the consequences of failing my English class.	1	2	3	4	5
22. I don't understand why some people get so upset over English classes.	1	2	3	4	5
23. In English class, I can get so nervous I forget things I know.	1	2	3	4	5
24. It embarrasses me to volunteer answers in English class.	1	2	3	4	5
25. I get upset when I don't understand what the teacher is correcting.	1	2	3	4	5
26. Even if I am well-prepared for English class, I feel anxious about it.	1	2	3	4	5
27. I often feel like not going to my English class.	1	2	3	4	5
28. I feel confident when I speak in English class.	1	2	3	4	5
29. I am afraid that my English teacher is ready to correct every mistake I make.	1	2	3	4	5
30. I can feel my heart pounding when I'm going to be called on in English class.	1	2	3	4	5
31. The more I study for an English test, the more confused I get.	1	2	3	4	5
32. I don't feel pressure to be well-prepared for my English class.	1	2	3	4	5
33. I always feel that the other students speak English better than I do.	1	2	3	4	5
34. I feel very self-conscious about speaking English in front of other students.	1	2	3	4	5
35. English class moves so quickly I worry about getting left behind.	1	2	3	4	5
36. I feel more tense and nervous in my English class than in my other classes.	1	2	3	4	5
37. I get nervous and confused when I am speaking in myEnglish class.	1	2	3	4	5
38. When I'm on my way to English class, I feel very confident and relaxed.	1	2	3	4	5
39. I get nervous when I don't understand every word theEnglish teacher says.	1	2	3	4	5
40. I feel overwhelmed by the number of rules you have to learn to speak English.	1	2	3	4	5
41. I am afraid that the other students will laugh at me when I speak English.	1	2	3	4	5
42. I get nervous when the English teacher asks questions that I haven't prepared in advance.	1	2	3	4	5
